# Strengthening Routine Data Systems to Track the HIV Epidemic and Guide the Response in Sub-Saharan Africa

**DOI:** 10.2196/publichealth.9344

**Published:** 2018-04-03

**Authors:** Brian Rice, Andrew Boulle, Stefan Baral, Matthias Egger, Paul Mee, Elizabeth Fearon, Georges Reniers, Jim Todd, Sandra Schwarcz, Sharon Weir, George Rutherford, James Hargreaves

**Affiliations:** ^1^ Social and Environmental Health Research Faculty of Public Health and Policy London School of Hygiene and Tropical Medicine London United Kingdom; ^2^ Centre for Infectious Disease Epidemiology and Research Faculty of Health Sciences University of Cape Town Cape Town South Africa; ^3^ Department of Epidemiology Johns Hopkins School of Public Health Baltimore, MD United States; ^4^ Institute of Social and Preventive Medicine University of Bern Bern Switzerland; ^5^ Department of Population Health Faculty of Epidemiology and Population Health London School of Hygiene and Tropical Medicine London United Kingdom; ^6^ Global Health Sciences University of California San Francisco San Francisco, CA United States; ^7^ Department of Epidemiology University of North Carolina Chapel Hill, NC United States

**Keywords:** HIV, data, systems, surveillance, sub-Saharan Africa, testing, treatment, prevention, cascade, monitoring, quality assessment, clinical, program

## Abstract

The global HIV response has entered a new phase with the recommendation of treating all persons living with HIV with antiretroviral therapy, and with the goals of reducing new infections and AIDS-related deaths to fewer than 500,000 by 2020. This new phase has intensive data requirements that will need to utilize routine data collected through service delivery platforms to monitor progress toward these goals. With a focus on sub-Saharan African, we present the following priorities to improve the demand, supply, and use of routine HIV data: (1) strengthening patient-level HIV data systems that support continuity of clinical care and document sentinel events; (2) leveraging data from HIV testing programs; (3) using targeting data collection in communities and among clients; and (4) building capacity and promoting a culture of HIV data quality assessment and use. When fully leveraged, routine data can efficiently provide timely information at a local level to inform action, as well as provide information at scale with wide geographic coverage to strengthen estimation efforts.

## Background

The global HIV response has entered a new phase with the recommendation of treating all persons living with HIV with antiretroviral therapy [1], and with the goals of reducing the number of new HIV infections and AIDS-related deaths to fewer than 500,000 by 2020 [2]. This new phase has intensive data requirements to monitor progress toward these goals.

The infrastructure of national-level public health surveillance platforms across the most heavily impacted countries, particularly in sub-Saharan Africa, is improving. This presents an opportunity for a major shift in the type of data used to inform country-level HIV prevention, treatment, and care responses. Central to this shift are recommendations by the World Health Organization (WHO) for investing in strategies to collect and leverage routine HIV data to track the HIV epidemic and ultimately, the response [3]. Moreover, new laboratory technologies, the growth of networked information systems, and the development of sophisticated data collection and analysis methods offer the potential to develop better strategic information.

Concurrent with these advances are shifts away from the mainstays of HIV. It has been suggested that the ethical underpinnings of unlinked anonymous HIV testing of pregnant women in sentinel surveillance have weakened, particularly in areas where HIV status information may be extracted from routine medical records [4-6]. National household-level surveys require large investments and provide only periodic data on which to anchor trend estimates. There remains a widespread absence of HIV incidence data at a time when the interpretation of prevalence trends is becoming ever more complex as treatment continues to expand and survival continues to lengthen. Intensive community-based cohort studies provide critical insights on epidemic dynamics but only over a limited geographic scope [7]. Sentinel HIV treatment cohorts and clinical databases are compromised by challenges in ascertaining outcomes and ensuring follow-up [8].

To promote sustainability, operational and surveillance platforms that utilize the latest computer technology for data storage are required. These platforms will ideally provide information in a timely fashion and at a scale and degree of detail that will improve our understanding of the distributions of HIV risk and determinants of that risk across settings. Similarly, we need surveillance systems that can monitor progress towards reducing the undiagnosed fraction of persons living with HIV, accelerate their pathway to care, and support retention in care. A greater focus on strengthening the demand, supply, analysis, and use of routine HIV data is essential to meet these needs and guide prevention, testing, and treatment efforts.

Focusing on sub-Saharan Africa, we present the following priorities for action to improve routine HIV data: (1) strengthening individualized HIV data systems that support continuity of clinical care and document sentinel events; (2) leveraging data from HIV testing programs; (3) promoting targeted routine data collection in communities and among clients; and (4) building capacity and promoting a culture of HIV data quality assessment and use (Multimedia Appendix 1). We define routine data as data generated, through regular procedure, from service-delivery platforms for prevention, testing and treatment, community and client surveys designed to inform to inform programmatic or strategic needs, and national registries. These data may be paper-based or electronic (preferably electronic) and presented aggregately or individually (preferably individually). All of the surveillance data recommended in this paper can and should be collected routinely.

## Priorities for Action

### Strengthen Individualized HIV Data Systems

Although the HIV care continuum [9-12] and 90-90-90 fast-track strategy [13] have given energy to measuring progress and driving scale-up in treatment programs, there remains much to do to ensure progress is accurately tracked. In many sub-Saharan African countries, patient monitoring data come from multiple sources, are of varying quality, and are often recorded electronically long after they have been combined to create aggregate reports. As these systems frequently monitor a person’s care pathway only from commencement of treatment, the pathway from HIV infection to diagnosis and to treatment is poorly understood, as is the wider clinical experience. Most current district health information systems rely on aggregate data making it difficult to assess how complete or accurate the data are or whether duplicate records are present [14].

#### Track Patients Within and Across Programs

Central to maximizing the utility of routine data is the idea of patient tracking. Data systems that track individuals over time and space are the only practicable way to ensure good clinical care and program responses. The ability to link individuals in this way enables the identification of persons lost to follow-up from one facility who have silently transferred to another facility, or who have died in the community, thereby informing targeted patient tracing or back-to-care programs [15]. These same systems also offer invaluable opportunities for strategic information based on de-duplicated individual-level records to compliment traditional reporting through management information systems in which the data are aggregated at source, and improve national and global estimates.

Individual patient data leveraged from multiple systems can provide an important data source for developing a comprehensive strategic HIV information system, such as case surveillance [3,14]. To expand existing HIV surveillance activities, the WHO has recommended adopting or strengthening such systems [3]. When collated through a comprehensive strategic HIV information system these patient data can provide a count of HIV infected and diagnosed individuals across a specified geographic area, and provide a comprehensive and timely picture of the care cascade. A comprehensive strategic HIV information system is but one component among a suite of components to strengthen the use of patient data in surveillance. Wherever possible, extracting data from systems already in place for operational and clinical purposes is preferred to requiring clinicians to additionally report events for surveillance purposes.

#### Develop Unique Universal Personal Identifiers

To facilitate system linkage, better methods are required for the development and integration of unique, universal, health identifiers that work across multiple health services and systems, such as linkage to vital registration, tuberculosis and viral hepatitis control programs, and maternal, new-born, and child health systems [3,8,16,17]. Lessons learned from countries with substantial experience with unique identifiers should be considered to inform adoption or expansion in other countries.

Approaches may include civil identification numbers, national health identifiers or master patient indexes (a database used by multiple locations to consistently maintain information on each registered patient ), or developing bespoke matching algorithms to link records based on existing patient identifying information (eg, sex, age, and a representation of name such as a Soundex) [18]. In developing unique identifiers, it is essential to consult with and address the concerns of clients and civil society on the potential for confidentiality breaches and consequent human rights abuses.

### Utilize Data From HIV Testing Platforms

The growing array of mechanisms for delivering HIV testing in clinical and non-clinical settings offers great potential for epidemiological intelligence. However, realizing this potential requires careful thought and investment.

#### Re-Focus on Data Systems for New Diagnoses

Data collected through HIV testing platforms, both negative and positive results, can inform prevention and clinical service provision. Site-specific information on newly diagnosed persons, including sex, age (as derived from date of birth), CD4 cell count and, where feasible, probable route of infection and a marker of residence, can be used to identify geographical regions and groups where HIV incidence is elevated. Spatial maps can be produced from these data to inform the provision of targeted and to-scale prevention and treatment services. Information on persons testing HIV negative can be utilized to inform appropriate prevention service provision. Developing data quality assurance protocols can help ensure the HIV testing data we collect are comprehensive and robust.

Testing data are critical for monitoring early (CD4 cell count greater than 350 cells/µL) and, conversely, late diagnosis (less than 350 cells/µL). As low CD4 cell counts can be triggers for more intensive follow-up [19], testing data can be used to monitor differentiated care models and promote earlier diagnosis. Testing data are also critical for monitoring the pathway from diagnosis to care, an important and often ignored step in the care cascade. In addition, to objectively monitor performance across the 90-90-90 targets, a first stage denominator of overall HIV prevalence is required. To monitor the first and second 90s we require an estimate of persons living with HIV who have been diagnosed for the numerator and denominator, respectively. If systems are strengthened, routine testing data should in the future directly provide estimates of diagnosed HIV, and indirectly through modeling, of undiagnosed HIV in sub-Saharan African settings.

#### Incorporate Data from Multiple Testing Platforms

Data collected through facility, community, and home-based testing platforms, key population outreach programs and, increasingly, through the expansion of self-testing all offer the potential for informing action. To utilize these data, a better understanding of the impact of shifting combinations, and increased coverage, of testing provision on reducing the number of previously untested persons and the number undiagnosed is needed, as is gaining a better understanding of the drivers for testing outside of traditional clinical settings.

The ongoing shift for surveillance purposes from unlinked anonymous testing in antenatal sentinel sites to using routine antenatal testing data collected through existing HIV program monitoring systems provides an opportunity to improve the level of detail by which HIV prevalence estimates are presented [20,21]. If coverage of HIV testing services at antenatal clinics is high, and if the available data are of high quality, this shift can improve the representativeness of HIV prevalence estimates as they are among all pregnant women rather than just among women from a non-representative sample of health facilities. However, there is a need to conduct quality assurance assessments of these data as there are remaining concerns that routine data, as compared to sentinel site data, may present greater variation in coverage and quality [22-24].

The expanding number of mechanisms for routinely delivering HIV tests ensures methodological improvements are required to appropriately analyze and interpret the data generated at the local and national level. It is likely that, amongst others, care-seeking patterns, existing knowledge of HIV status, test-kit quality, missing patient responses, and a variable prevalence of electronic data systems and standardized practices will influence estimates.

#### Employ Recency Assays

Understanding where and among whom new infections are occurring is critical to plan programs and to track the incidence of new infections over time to assess whether control measures are working. To date, most estimates of HIV incidence in sub-Saharan Africa come from modeling efforts based on trends in HIV prevalence, births and deaths, and information about the coverage of treatment programs. In some settings estimates that do not rely on modeling are based on community cohort studies with repeat HIV testing, although this has rarely, with some exceptions [25], been done at national scale.

Recent infection testing algorithms (RITA) can distinguish recently acquired infection from long-standing infection [26,27]. Recency testing offers the potential to inform action for individual patients, their networks and programs and, if incorporated into routine testing and programmatic surveys, to strengthen estimates of incidence. Examples might include identifying clusters of infection among key populations in outreach testing platforms, or complimenting data on HIV prevalence in routine testing of pregnant women with information on recency. Attention to test accuracy, data quality, ethics, sample size, and analysis and interpretation will be necessary. Nevertheless, the wider adoption of these technologies within routine data systems, especially if the price can be reduced, has the potential to bring new strategic information.

### Targeted Routine Data Collection Among Communities and Clients

Strategically deployed community-based and client surveys (eg, tracing studies) can routinely be conducted to complement our understanding gleaned through prevention, testing, and treatment programs. They can inform and correct some of the biases of routine programmatic data, and provide information about individuals and populations at risk of infection or treatment failure, the scale and nature of their needs, and potential barriers to the effectiveness of prevention programs [28]. These surveys are often cost efficient compared to population-wide surveys and provide an opportunity to contextualize populations represented within routinely collected service data, and to develop HIV prevention cascades to guide prevention programs [29,30]. Because these populations are often hidden and stigmatized, and may practice, in some cases, illegal behaviors, novel methods are required.

#### Systematize Key Population Surveillance

In sub-Saharan Africa, venue-based and respondent-driven sampling strategies are commonly used to characterize key populations for whom there is no practical sampling frame. Venue-based sampling focuses on settings where key populations congregate, while respondent-driven sampling leverages networks of people sharing risk characteristics [31-33]. Although widely used in Africa, these approaches have not to date received the same level of attention as national-level general population surveys.

These sampling methods tend to be based in local sites and often have not been designed to be representative at larger scale. Current practice has led to a range of funders and programs deploying a variety of sampling methods. Heterogeneity in methods and sample populations limit the potential for the data to be used to track trends or be incorporated in modeling efforts designed to inform resource allocation decisions.

The implications of which sampling approach is used has been shown to be significant, with the characteristics of the populations recruited differing [33]. Through gaining a greater understanding of the strengths, limitations, and interpretation of sampling methods, a more systematic approach to their deployment can be developed. Decision-trees can be adopted to highlight which assumptions were met, which were not, and how best to proceed given different circumstances and statistical tools developed to derive best estimates from data arising from routine services and targeted surveys.

#### Identify Populations With Greatest Needs and Develop Tools to Guide Service Provision

Surveillance for program improvement requires identifying the location, needs and size of populations at high risk, and recognizing individuals may move in and out of periods of high risk and/or fall under one or more high-risk behavioral group. It is essential that program decision-making is based not only on those reached by services but also those unreached.

There is a growing call for community-based efforts that differentiate sub-groups and risk to inform targeted service provision. Within the Determined, Resilient, Empowered, AIDS-Free, Mentored, and Safe Women (DREAMS) initiative, the Girl Roster tool is being deployed to help reach adolescent girls in need, while variants of network-based referral can also be used to identify young women who sell or trade sex for referral into programs; approaches such as peer-led micro-planning can be deployed to strengthen programming [34-36].

### Build Capacity and Promote a Culture of HIV Data Quality Assessment and Use

As good local data spur action locally and create better data for onward reporting, the systems designed ought to be sustainable and responsive to the local context and produce data that are user-friendly, sustainable, and meet the needs of stakeholders at various levels. Long-term investment in human and technological capacity is required so that people within highly impacted countries can create their own solutions and develop sustainable practice. Collectively, we should avoid demanding more data unless this can be derived without impact on data collectors or front-line clinicians.

#### Encourage Responsibility for Data quality at the Local Level

Data quality at the local-level can be improved through promoting timely, comprehensive and accurate facility-based record keeping, matching data needs to setting, and building buy-in and trust among stakeholders. Engagement with and training of frontline data staff are critical. Understanding the needs of clinical staff is also critical. If data systems are strengthened for clinical care governance, data quality and strategic information will benefit. Deriving population-representative strategic data from routine clinical and/or health systems will also reduce the potential for data collection for surveillance purposes becoming a separate resource-intensive activity in itself.

#### Evaluate Data Quality

At the sub-national and national level, a standardized approach to system design, reducing redundancy, and limiting the development of parallel systems (systems with a shared purpose developed independent of one another) should be encouraged. Through reviewing existing systems and/or surveillance frameworks [37,38], elements of transferable practice can be identified to inform system design and the development of training materials.

The engagement and training of data stewards at the sub-national and national-level (eg, district and national Minister of Health staff) to oversee programmatic, clinical, and surveillance activities are critical. These custodians can promote robust feedback loops so that the common problem of data travelling only in one direction, from facility to health departments in aggregate form, can be avoided. Feedback to those who collect, collate, and report data should rank reporting sites according to data quality and program outcomes. Feedback should incorporate the use of maps and dashboards, and make clear what the benefits of using the data provided have been (eg, informing policy and interventions).

To ensure data outputs are interpreted correctly, assessments of data sources are required to indicate quality and value. For example, to compare results across populations and time for each stage of the care cascade, a traffic light system could be used to indicate confidence levels in data sources of high, medium, and low. To generate high quality data, and promote accurate interpretation, conducting quality assessments and analyses to investigate and correct some of the biases of routine data is also needed. Bias may arise, from missing data, non-representativeness, double counting (aggregate data particularly suffer from this), or loss to follow-up. Through identifying bias, methods such as adjustment factors, to support the interpretation of routine data outputs, can be designed.

#### Develop Procedural Documents

At all levels, standard operational procedures for data collection and quality assurance should be developed, promoted, and updated regularly. These procedural documents can specify how routine data quality assessments are to be conducted and instruct how identified errors are addressed. They can stipulate standardized analytical methods for producing useful programmatic, clinical and surveillance outputs that account for potential biases and include accurate assessments of error, uncertainty, and explicit consideration of important assumptions. These documents can also provide guidance for conducting regular output evaluations to ensure accuracy, to ensure the needs of users are being met, and to monitor impact.

#### Consider Timeliness of HIV Data Cycles

Data cycles need to be feasible and designed to meet the different needs of a variety of stakeholders. For clinical activities, it is required that data be collected, reviewed, and shared as close to real time as possible. Such provision of data can enable missed appointments to be acted upon immediately. For surveillance activities, data producers will ideally collate as few variables as necessary, and only those that inform action. They will also assess the minimum period required for conducting comprehensive data collection and quality improvement, and producing and reviewing meaningful outputs. In conjunction with this, data users should assess the timeframes within which they can receive, review, and use the data for program improvement. The development and endorsement of standard operating procedures can guide these efforts.

## Discussion

To reduce HIV incidence and mortality in sub-Saharan Africa we need to provide effective prevention methods, identify all individuals living with undiagnosed HIV infection, improve the pathway between diagnosis and care, retain people once they are in care, and promote antiretroviral therapy adherence. To achieve this a new phase must be entered, one where we innovate and improve the routine data systems that support HIV prevention and care and where we shift from using routine data purely for descriptive purposes towards directly informing and improving clinical care and program performance. Robust data to inform advocacy, to secure resources, and to create an environment conducive to better rights and fit within national monitoring and evaluation policies are required.

Here, we presented four priorities that will result in durable and replicable data systems that collect, store, and produce individual-level data, and that give rise to user-friendly tools that can be used by local, national, and international practitioners to drive more effective and efficient clinical management and prevention and care programming. We focus on routine data that are, or might be, collected in sub-Saharan Africa as part of service delivery requirements. We are united in our appreciation of the value of national surveys of HIV prevalence and incidence, community-based cohort studies, and research studies and trials, but our focus is now on routine HIV data systems that will be central to the next wave of HIV strategic information. While we focused on HIV-specific data, we also recognize the enormous value of strengthening vital registration and broader health information systems.

In promoting the use of routine data we need to assess the level of disaggregation useful to inform action and recognize that uncertainty and/or imprecision increases with the level of disaggregation. We also need to collect and present data by geographies that map to how programs and services are delivered.

When fully leveraged, routine data can efficiently provide timely information at a local level to inform action, as well as provide information at scale with wide geographic coverage to strengthen estimation efforts. To maximize the use of these data we need to overcome complex challenges. We need to promote sustainability through investment in data system design, implementation and capacity, and to ensure the data we produce are credible and valuable through strong attention to data quality and use, and through careful analysis and investigation of potential biases.

## Appendix

Multimedia Appendix 1 Summary of priorities for action.

## Abbreviations

WHO: World Health Organization
MeSH: Measurement and Surveillance of HIV Epidemics
