# Supplementary material for: Strengthening Routine Data Systems to Track the HIV Epidemic and Guide the Response in Sub-Saharan Africa
Source: JMIR Public Health Surveill. 2018 Apr 3;4(2):e36. doi: 10.2196/publichealth.9344 (PMC5904448; doi:10.2196/publichealth.9344)
Supplement: Multimedia Appendix 1 [file publichealth_v4i2e36_app1.pdf]

Priorities	Recommendations	Exemplar
Strengthen individualized HIV data systems that support continuity of clinical care and document sentinel events	Track patients within and across programs	Develop case surveillance
	Develop unique universal personal identifiers whilst ensuring confidentiality	Develop / implement civil identification numbers, national health identifiers and master patient indexes
Utilize data from HIV testing platforms	Re-focus on data systems for new diagnoses	Utilize site specific routine testing data
	Incorporate data from multiple testing platforms	Exploit facility, community and home-based testing platforms, key population outreach and self-testing programs
	Employ recency assays	Pilot test in a range of service-provision contexts including PMTCT, testing and counseling facilities, and outreach testing services
Targeted routine data collection among communities and clients	Systematize key population surveillance	Assess strengths and limitations of different sampling methods
	Identify populations with greatest needs and develop tools to guide service provision	Implement community-based surveys that appraise risk across sub-groups
Build capacity and promote a culture of HIV data quality assessment and use	Encourage responsibility for data quality at the local level	Engage frontline data staff in promoting effective record keeping
	Evaluate data quality at the sub-national, national and global level	Develop and promote robust data quality feedback loops in existing systems of reporting between facilities and health departments
	Develop procedural documents	Specify processes for conducting ongoing data quality assessment and for dealing with errors
	Consider timeliness of HIV data cycles	For surveillance activities identify the minimum period required for conducting data collection and quality improvement, and producing and reviewing meaningful outputs
